# Rare congenital absence of tail (anury) and anus (atresia ani) in male camel (*Camelus dromedarius*) calf

**Published:** 2012-06-30

**Authors:** S. Anwar, G.N. Purohit

**Affiliations:** 1 *Al Qattara Veterinary Hospital, Al Ain, UAE*; 2 *Department of Veterinary Gynecology and Obstetrics, College of Veterinary and Animal Science, Rajasthan University of Veterinary and Animal Sciences, Bikaner, Rajasthan, 334001, India*

**Keywords:** Anury, Atresia ani, Camel, Taillessness

## Abstract

A one-day old male camel calf was presented to the Al-Qattara Veterinary Hospital with complaints of abdominal straining and lack of defecation. On examination it was found that the calf had no tail, the posterior sacral margin was blunt and the anal opening was absent. The case was diagnosed as congenital anury with atresia ani. The animal was sedated with 0.1 mg/kg of xylazine administered intramuscularly and under local infiltration with 2% lidocaine a circular incision was made at the anal area to create an anal opening. The animal passed plenty of meconium. The cut edges were sutured with horizontal mattress sutures. The animal was administered penicillin and streptomycin for 5 days and had an uneventful recovery. It is reported that congenital anury rarely occurs in one humped camel and accompanied atresia ani can be surgically treated.

## Introduction

Congenital taillessness is a rare defect described for many breeds of cattle (Huston and Weardon, 1958; Ayers *et al.*, 1989; Belge *et al.*, 2000; Ingham and Widdows, 2005; Lotfi and Shahryar, 2009; Debasis and Mousumi, 2010), dogs (Pullig, 1953; Hall *et al.*, 1987; Cho and Kim, 2006; Cho *et al.*, 2008; Hytonen *et al.*, 2009), rats (Chesley and Dunn, 1936; Dunn *et al.*, 1942) and domestic fowl (Dunn, 1925). The condition has been rarely described in goat (Debasis and Mousumi, 2009) and sheep (Basrur and Yadav, 1990; Mahmood *et al.*, 2001).

Tail abnormalities are sometimes associated with rectal adhesions and excretion difficulties (Huston and Weardon, 1958; Mahmood *et al.*, 2001; Debasis and Mousumi, 2009) and necessitate surgical correction when the young ones are born with conditions like atresia ani (Mahmood *et al.*, 2001; Suthar *et al.*, 2010). The congenital caudal vertebral malformations have been described in the alpaca (Vaughan *et al.*, 2000) but no case of anury was recorded. In the present report we describe a case of tailless male camel calf born with atresia ani and its surgical correction.

## Case History

One-day old male camel calf was presented to Al Qattara Veterinary Hospital with a complaint of not passing feces. The signs of tenesmus and abdominal pain were observed and slight distension of the abdomen was evident.

**Fig. 1:**
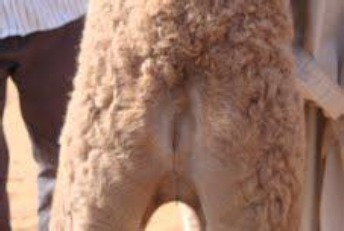
Caudal end of one-day old camel with atresia ani.

**Fig. 2:**
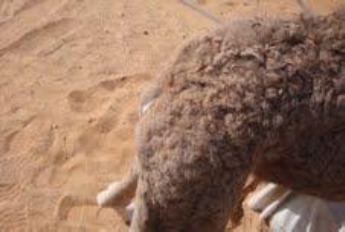
Lateral view of one-day old camel showing anury.

Up on examination it was found that the calf had no tail and the opening of anus was closed with a scar at the site of opening (Figures 1 and 2). Physically the calf was walking normally and there was no sign of urinary incontinence.

The coat color was light brown and the hair was normal. On gross examination there was no evidence of tail, the posterior sacral margin was blunt. The dam of the calf was native breed of Al Ain area in its third parity and had no history of illness except retention of placenta following this birth. Both the dam (mother) and the sire (father) of the calf were having normal tails. Gross and physical examination confirmed the case as atresia ani with tailless congenital abnormality.

## Surgical technique

The calf was sedated with xylazine hydrochloride (Rompun, Bayer Animal Health Germany) at a dose rate of 0.1mg/kg body weight (IM) and was controlled in lateral recumbency. The perineal area was prepared for aseptic surgery by scrubbing with povidone iodine. The site was anesthetized by infiltration of 5 ml of 2% lidocaine hydrochloride subcutaneously at the proposed site of incision around the scar below the sacral margin. The skin at the scar was slightly pulled backward and a circular piece slightly larger than the normal anal opening was removed.

**Fig. 3:**
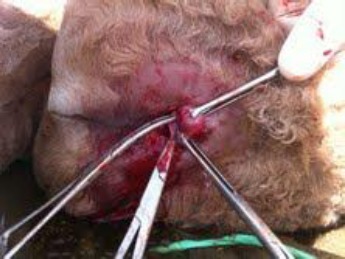
A one-day old camel during surgery.

**Fig. 4:**
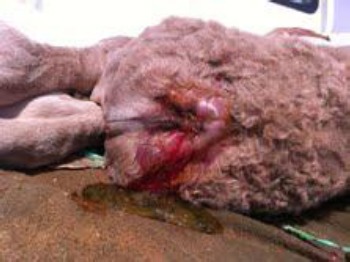
Surgical correction of atresia ani (Note presence of meconium on the ground).

Careful dissection was carried out further inwardly avoiding adjacent muscles. The blind end of anal canal (ampula recti) was caught and cut with scissors. Immediately, meconium started flowing out. When meconium flow became minimal, the cut edges of the skin were sutured with the cut edge of the anal opening all around (360 degrees) with horizontal mattress sutures using USP 0 non absorbable (chromic catgut 2/0) suture material keeping the knot outside (Figures 3 and 4).

## Post-operative care

The surgical wound was cleaned and dressed regularly with liquid povidone iodine. Penicillin and Streptomycin suspension (Pen & Strep, Norbrook, UK) was administrated at a dose of 1 ml/kg for 5 days intramuscularly, to prevent infection. Skin sutures were removed on the 10^th^ post-operative day.

## Discussion

The taillessness condition has been known to have a hereditary or congenital origin. The genetic basis of tailless mice has been a focus of many studies which showed that tailless mice contained two alleles, each of which was lethal when homozygous resulting into anury (Chesley and Dunn, 1936; Gruneberg, 1958; Wilson *et al.*, 1995; Kilic, 2004).

Short tailed dogs are present in many breeds but the T gene mutation has been known to result in short tails in the Pembroke Welsh Corgis (Indrebo *et al.*, 2008). The T gene mutation can cause anury and Brachury (short tails), but the T gene mutation is not present in all breeds of dogs suggesting other genetic or congenital factors affecting tail phenotype in dogs (Hytonen *et al.*, 2009).

In cattle most tail defects are considered to have genetic origins (Schalles and Cundiff, 1999; Belge *et al.*, 2000) and these abnormalities mostly appear in the process of crossing different breeds (Hills, 1997; Bahr and Distl, 2005).

Atresia ani is also considered a defective development that is sex linked in sheep (Dennis and Leipold, 1972) and due to an autosomal recessive gene in pigs (Harkin *et al.*, 1982). Congenital defects may arise because of many factors including ingestion of some toxic plants, effects of some infectious agents and administration of some drugs (Rosseaux and Ribble 1988). The cause of anomalous development may occasionally be obvious but more often is obscure because of its multifactorial nature (Rosseaux and Ribble, 1988).

The reason for anury in the present case could not be ascertained. The male to which the camel calf was born had not sired similar calves previously. It has been mentioned that the probability of occurrence of anury in female calves of dairy cattle is twice the probability of occurrence in male calves (Lotfi and Shahryar, 2009). However; the sex of the camel calf presently was male.

The present camel calf had anury along with atresia ani. Defects of the rectum including atresia ani may occur with anury (Huston and Weardon, 1958; Debasis and Mousumi, 2009; Mahmood *et al.*, 2001; Varghese *et al.*, 2010).

Surgical management of atresia ani was simple surgical excision followed by making a mucocutaneous junction. Atresia ani has been recorded in alpaca (Del Campo *et al.*, 1993) and in llama (Whitehead, 2009) but similar case was not reported.

Calves are referred for atresia ani because of fecal retention (Suthar *et al.*, 2010; Varghese *et al.*, 2010) and similar problem was seen in the day old camel calf in the present report. A gush of meconium passes out on first surgical opening of congenitally closed rectal wall at the anus. The healing was uneventful and the calf had no subsequent problem.

It is reported that congenital anury with atresia ani rarely occurs in camels and surgical correction of atresia ani is mandatory.
